# Novel treatments for complications after bariatric surgery

**DOI:** 10.1186/s13022-015-0021-2

**Published:** 2016-03-15

**Authors:** Julián Hernández, Camilo Boza

**Affiliations:** Department of Digestive Surgery, Pontificia Universidad Católica De Chile, Santiago, Chile; Bariatric Surgery Unit, Department of Digestive Surgery, Clínica Las Condes, Estoril 450, Las Condes, Santiago, Chile

## Abstract

Bariatric surgery has been considered one of best treatments for obesity. As every surgical procedure—and any medical intervention, it is not exempt of complications, among which leaks, strictures, acute hemorrhages and fistulae highlight. Leaks are more common in the gastro-jejunal anastomosis (GJA) in the case of Roux-en-y Gastric Bypass (RYGB), while in Sleeve Gastrectomy (LSG) they locate in the stapler line. Stenosis can be seen in the gastro-jejunostomy in the RYGB and in the gastric tube in case of the LSG. For each of these complications, many innovative solutions have been developed, including new surgical devices. In spite of promising good results, evidence regarding utility and safeness of these technologies is still scarce. Self-expandable endoscopic stents have been used to treat leaks, with an overall success rate of 80–90 % and a migration rate of 15–35 %. The bear trap-like over-the-scope (Ovesco) clips have been used to treat GI hemorrhages, leaks and even fistulae, with a 70–80 % success rate, although more endoscopic sessions may be needed. Overstitch, an endosurgical suture devices, have been used to treat leaks, fistulae and perforations. Overall, technical success achievement approaches to 90 %, while clinical success ranges from 80 to 90 %, except for leaks closure, where a lower success rate has been observed. Despite of all of these advances, early diagnosis and treatment remains the main strategy to achieve success. In summary, novel therapies for complication management can be very useful, though further studies with larger series are still needed in order to confirm their efficacy and safeness.

## Background

Bariatric surgery has become an accepted treatment for obesity in the last decades. Obesity and its comorbidities can be reasonably treated with many types of bariatric procedures. Along with obesity prevalence, bariatric surgery has increased in number and type. Currently they are one of the most frequent elective surgeries performed. Types of bariatric procedures are diverse; however, the commonest are laparoscopic sleeve gastrectomy (LSG), Roux-en-Y gastric bypass (RYGB) and adjustable gastric band (AGB). Less common are biliopancreatic diversion with duodenal switch (BPD-DS) and intragastric balloon (IB).

Complications in bariatric surgery can be observed a many stages after the intervention. Mechanisms involved seem to have their origins in surgical techniques (based on the surgeon’s experience), isquemia, use of drugs and the presence of comorbidities. Management of these complications have evolved in the last years due to technology advances to better results, however, as most medical problems, early diagnosis and treatment are still the most important prognostic factors.

## Bariatric surgery complications

Excepting a few cases, most complications are common to all bariatric procedures. According to Su-Hsin Chang et al. [1] in a systematic review and meta-analysis published in JAMA Surgery last year, with more than 160,000 patients, overall postoperative bariatric complication rates range from 10 to 17 % and reoperation rates approximately 7 %. Nonetheless, mortality still remains low, from 0.08 to 0.35 %. Although there are many types of complications, Table 1 shows the most frequent ones.

**Table 1:** Most frequent bariatric surgery complications

Leaks
Acute hemorrhages
Stenosis
Bowel obstruction
Marginal ulcers
Fistula
Incisional hernia
GERD

### Leaks

Postsurgical leaks can have their origin in gastrojejunal anastomosis in the case of RYGB or the staple line in LSG. In the latter case, they can be classified as acute, early, late and chronic (<1, 1–6, 6–12 and >12 weeks, respectively) [2]. According to most recent evidence, in experienced institutions LSG leaks border 1 % incidence [3] and according to Parikh et al. [4] in a systematic review and meta-analysis of 9991 patients in 2013, leaks rate in a general overview after LSG is 2.2 %. After RYGB, it ranges from 1.68 to 2.05 %.

Diagnosis can be challenging and any delay of the treatment worsens patient’s prognosis. Morbidity mostly expresses as abdominal pain, taquicardia. Sometimes as an abdominal abscess, sepsis or chronic fistula, all of which are serious complications that usually lead to intensive care units admission, longer hospital stay and the development of other medical complications. Risk factors associated with postoperative leaks are age, BMI, male sex, type 2 diabetes mellitus, obstructive sleep apnea syndrome and hypertension [5].

Management of postoperative leaks can be really complex and it can be conservative or invasive depending on the patient’s hemodynamic stability and infectious status, but as a general rule, life-threatening or chronic events require surgical interventions, otherwise conservative management is preferred.

As most complications, early diagnosis is critical to prevent evolution to general sepsis and peritonitis.

### Acute hemorrhages

Hemorrhages usually have their origin in staple line, but they can also come from anastomotic or gastric remnant ulcers. In the former case, there is wide agreement that oversewing the staple line helps preventing this complication.

Patient’s medical history gains importance in this issue since comorbidities such as coagulopathies and anti-hemostatic drugs (warfarin, acenocumarol, ASA) transports the medical team to a totally different scenario. A complete anamnesis is therefore of great importance.

Management consists in conservative support and endoscopic interventions in the majority of cases, but hemorrhages of big quantity (with hemodynamic instability) or from sites inaccessible to endoscope require surgical intervention.

### Stenosis

Stenosis refers to stricture of an anatomical duct or cavity. Therefore, in bariatric surgery there are several sites of possible narrowing, but mainly two: in the RYGB the gastrojejunal anastomosis and in LSG the gastric remnant. They develop gradually so they belong to late complications; early strictures in LSG become symptomatic hardly before 6 weeks, while in RYGB they do in the late third or early fourth week.

Gastrojejunal stenosis is a relative frequent complication, with incidences varying from 4 to 27 % depending on the clinical series described. In the other hand, gastric stenoses border 1 % incidence and their apparition is associated with the smaller size of the bougie used.

Clinical manifestations of stenosis include food intolerance, vomiting and dysphagia without abdominal pain. Diagnosis can be made through contrasted radiology or endoscopy, which can be therapeutic as well. As in other cases, management can be conservative, endoscopical or surgical.

### Bowel obstruction

Bowel obstructions can be early or late, often due to internal hernias after gastric bypass in the former case and due to intraperitoneal adhesions in the latter. They reach 2 and 3 % incidence, respectively. Again, depending on patient’s stability and clinical manifestations, a conservative treatment can be planned, but surgical management is sometimes necessary.

### Marginal ulcers

Most marginal ulcers occur within the first 12 months of gastric bypass. Their frequency is described up to 16 % of patients. Among risk factors is *Helicobacter pylori* infection, gastro-gastric fistula, use of NSAIDs, and ischemia.

Management is complex because medical therapy does not seem to be effective, surgery has high incidence of complications associated and recurrence has been reported up to 7.7 %.

### Fistulas

Although fistulas can appear along almost any site of the digestive tract after surgery, gastro-gastric fistulas are particularly worrisome complications after Roux-en-Y gastric bypass. Incidence has been reported in up to 1.2 % of cases [6].

Clinical symptoms are mostly related to epigastric pain due to ulcerations around the anastomotic site. If asymptomatic, they can be managed conservatively at the beginning, but refractory pain and ulcers are the main indication for revisional surgery.

### Incisional hernia

This type of hernias can appear in any site of abdominal incision; however, its frequency in laparoscopic procedures, less than 1 %, differs greatly from open Roux-en-Y gastric bypass, around 8 %. Though this kind of intervention is every time less performed, complicated hernias have to be kept in mind because complications can come up suddenly years after the procedure.

### GERD

Gastro-esophageal reflux disease appears in approximately 12 % of cases after laparoscopic sleeve gastrectomy. Management includes dietary and lifestyle interventions along with pharmacotherapy, but sometimes revisional surgery is needed, of which gastric bypass shows to have the best resolution rate (around 56 % improvement in symptoms score). It is also necessary to rule out always the presence of hiatal hernia and repair it during revisional surgery.

### Gastric band complications

Adjustable gastric band is one the commonest procedures among bariatric surgery. Complications are mainly band slippage and gastric erosion. The former one causes severe dysphagia and vomiting in the early postoperative period, while the latter one’s manifestations, which can be as serious as GI hemorrhage or intra-abdominal infections, begin in a later setting.

## Diagnosis

As important as being up-to-date with the different diagnostic techniques available, is keeping clinical suspicion at a low threshold in every patient with a suggestive history of complications. Therefore, a detailed anamnesis and physical examination are crucial in order to detect abnormal courses of postoperative evolution. Early diagnosis prevents aggressive treatments and in many cases revisional procedures that involve high rates of morbidity. Most frequent clinical symptoms and signs are abdominal pain, tachycardia, nausea, vomiting, bloating and fever. Characteristics of abdominal pain should be taken into consideration.

The most frequent diagnostic procedures are contrasted studies along with abdominal (with or without pelvic) computed tomography. The former ones usually show upper GI leaks, fistulas and stenosis, while the latter ones detect intra-abdominal collections or intra-peritoneal bleeding. Endoscopy in the early postoperative period should be avoided, but if necessary it can diagnose and treat several complications, such as digestive hemorrhage.

## Management

Treatment should always consider, in the appropriate conditions, a conservative management. This includes adequate hydration, electrolyte homeostasis stabilization, nutritional support, oral or intravenous antibiotics, abscess drainage, etc. A correct decision of conservative management can avoid unnecessary surgical interventions, however, if it does not lead to a definitive resolution, it associates with longer hospital stays, patient’s discomfort and even the development of chronic leaks or fistulas.

### Surgery

Surgery is always the most complete procedure since it can assess the complete status of the previous surgery, and characteristics of the actual anatomy. Laparoscopic surgery has gained ground in the last 20 years along with all minimal-invasive procedures due to their lower incidence of postoperative infections among other reasons; however, it is not exempt of limitations, so the surgeon’s experience and surgical feasibility of laparoscopy have always to be weighed. Although extremely rare, when life-threatening events without clear explanation unexpectedly happen, open surgery is indicated.

### Self-expandable endoscopic stents

Self-expandable endoscopic stents (SEES) consist in endoluminal prostheses that seek the maintenance of digestive tract permeability. Traditionally, they have been used to treat benign stenosis and obstructions of neoplasic cause. In these cases, stents are usually made of metal, but more recently stents made of polyester with an inner coverage of silicon have appeared to treat different digestive complications, such as leaks and postoperative strictures. Metal stents have higher friction coefficient with digestive mucosa, thus, migration rates are lower than polyester ones. On the other hand, stronger fixation of metal stents determines greater difficulty in its extraction than polyester ones, due to tissue ingrowth under them and subsequent adhesion.

In recent years, SEES have been used to treat leaks detected after bariatric surgery. The mechanism would be to cover the site of the luminal defect, allowing it to heal without tissue injury. When they are used to treat strictures, stents permit to maintain cavities open, so that the healing process (cicatrization and fibrosis) finishes with a permeable duct.

Since stents were initially designed to be attached to the esophagus, when they are placed in the gastrojejunal anastomosis they tend to migrate more frequently.

In general, they are well tolerated. Some patients report nausea, dysphagia or discomfort and up to 9 % require revision, usually due to persistent leaks or migration. Enteral nutrition with liquid diet is mostly well tolerated. The clear advantage of this procedure is that this can be kept instead of parenteral nutrition with all the complications it carries.

When a stent fails to prevent leaks, it is not necessary to remove it. Instead, a second one can be placed over the first one, covering both proximal and distal edges. Regardless of this, stents should not remain in its place for more than 7 months. In leaks treatment, the highest proportion of patients keep it for 6 weeks, enough time to allow the wound to heal and not developing excessive adhesion to the tissue.

Evidence regarding stent use is scarce. One of the meta-analysis available assessed the use of stents postoperatively in 67 patients. They reported success in leakage management in 87.7 % of cases, migration in 17 % and successful extraction in 92 % [7]. In other studies, success in leakage management is approximately the same.

According to our experience, despite the long period needed to treat, the overall success rate of SEES use was 96 %. The migration rate was approximately 34 %, with the need of repositioning or replacement in the majority of these cases [8]. The experience of Schiesser et al. [2] follows this tendency with an 88 % success, though their management included other interventions such as Ovesco^®^ clips, discussed later.

Documented experiences in the use of SEES in chronic leaks or fistulas are rare and they generally reveal worse outcomes; recently, Puig et al. published their series with metal prostheses where only 4 out of 21 patients could be treated exclusively with SEES, though they concluded it may help suppress ongoing sepsis and allow patients to undergo oral nutrition before definitive surgical resolution [9].

Considering that stents avoid surgery with its morbidity and mortality, it is still an option that can be measured among others and a recent expert consensus considers it a valid option [10].

### Over-the-scope clips

This kind of endosurgical clips was recently introduced to treat GI hemorrhages and perforations. It consists in an applicator cap, a hand wheel and the clip. The applicator cap has three different diameters to fit in different endoscopes. The clips look like a bear trap, so they apply pressure equally in a circumference form. There are three different kinds of clips: one with blunt teeth and two with sharp teeth that differ from each other in their length. Blunt teeth clips are more often used to control bleeding, while the sharp ones are used to close perforations or to manage indurated, fibrotic tissue (usually associated with chronic injury).

To use this device it has to be fitted on the tip of the endoscope fixed over the applicator cap that incorporates a release thread which is passed through the working channel and connected to a turning wheel mounted on working channel access port. The main difference with other endosurgical clips is that in this case pressure is applied in a circumference form. They should not be used in sites adjacent to collections or infections.

As in other novel treatments, evidence is scarce. There have been cases reported with successful management of gastrointestinal or gastrocutaneous fistulas. The experience of Surace et al. with the biggest series reported, 19 patients with GI fistula showed a 74 % success [11].

Keren et al. [12] reported recently an 80 % success in treating postoperative leaks after LSG, in a series of 26 patients, with a median of three endoscopic sessions and no complications related to the procedure.

### Overstitch

The Overstitch system consists in a single-use endosurgical suture device which is attached to the endoscope and allows deploying various stitches in a relatively small working space. The curved needle design allows for controlled depth of suture placement and enables the placement of durable full-thickness stitches. It is capable of deploying and reloading sutures while maintaining direct visualization of the operative site, for better control and minimal scope insertions and removals.

The utility of this novel endoscopic suture system is that it can resolve several bariatric surgery complications: outlet and puch reduction, fistula closures, gastric placation and even GI bleeding. Evidence regarding Overstitch’s utility is scarce, being published mainly case reports. In one of the latest and biggest series ever published, Sharaiha et al. analyzed retrospectively 122 patients from eight different centers who were treated with Overstitch suturing system due to different causes, namely, fistulas, leaks, perforations and stent anchorage. A 97.5 % overall technical success was achieved, while clinical success for stent anchorage, perforations and anastomotic leak closure was 91.4, 93, 80 and 27 % [13].

### Stomaphyx

The Stompahyx system was created with the purpose of gastric plication. It places full-thickness polypropylene fasteners through the gastric mucosa, which is previously drawn into the device’s chamber by suction. The fasteners are not absorbable and are firm enough to maintain gastric plications. It can be used to reduce the gastric pouch or stoma in patients that have regained weight, but it can also be used to repair leaks in the GI wall.

Evidence regarding efficacy of Stomaphyx is scarce. Goyal et al. made a retrospective analysis of 53 patients post gastric bypass who underwent gastric pouch reduction with Stomaphyx, which showed EWL not sustained over time [14]. Consistently, the only randomized controlled trial comparing Stomaphyx with a sham endoscopic procedure in patients post RYGB with weight regain, performed by Eid et al. demonstrated clinically significant reduction in regained weight in only 22.2 % of participants, though this difference was significant with 3.4 % of sham procedure patients group [15]. Thus, Stomaphyx is a reasonable alternative in patients with weight regain in which the risks of a second surgery would exceed the benefits of weight loss, considering its limited reach.

### Endoscopic dilators

Stenosis after bariatric surgery has been traditionally treated with surgery or balloon dilation. Nowadays, Savary–Gilliard are the most widely used dilators in treating this condition. They consist in polyvinyl tubes with different diameters (5–20 mm) with a radio-opaque band to aid radiological localization. Benign and malignant esophageal stenosis can be resolved by this device, however, in bariatric surgery its utility is focused mainly in gastric stenosis post sleeve gastrectomy and anastomotic stenosis after Roux-en-Y gastric bypass.

Available evidence shows that anastomotic strictures can be resolved in around 75 % of cases through Savary-Gilliard dilators, with low morbidity and no mortality associated.

## Conclusion

Complications are the source of mortality after Bariatric Surgery. Early diagnosis and treatment remain the most important step to achieve good results. Novel treatments of complications have evolved to the arena of endoscopy. Any of these treatments should be indicated as soon as possible to promote better results. There has been limited results for chronic complications.
